# Synergistic Antitumor Interaction of Risedronate Sodium and Standard Anticancer Agents in Canine (D-17) and Human Osteosarcoma (U-2 OS) Cell Lines

**DOI:** 10.3390/ani12070866

**Published:** 2022-03-29

**Authors:** Dominik Poradowski, Aleksander Chrószcz, Bożena Obmińska-Mrukowicz

**Affiliations:** 1 Department of Animal Physiology and Biostructure, Division of Animal Anatomy, Faculty of Veterinary Medicine, Wrocław University of Environmental and Life Science, Kożuchowska 1, 51-631 Wroclaw, Poland; aleksander.chroszcz@upwr.edu.pl; 2 Department of Pharmacology and Toxicology, Division of Pharmacology, Faculty of Veterinary Medicine, Wrocław University of Environmental and Life Science, C. K. Norwida 31, 50-375 Wroclaw, Poland; bozena.obminska-mrukowicz@upwr.edu.pl

**Keywords:** risedronate sodium, cisplatin, carboplatin, doxorubicin, etoposide, interaction, canine and human *osteosarcoma* cell lines

## Abstract

**Simple Summary:**

The study investigated canine (D-17) and human (U-2 OS) *osteosarcoma* exposition to risedronate sodium and chosen standard anticancer drugs concurrently used in vitro. Risedronate sodium is frequently used in the therapy of bone tissue disorders. Our study demonstrated increased efficiency of cytostatic drugs in the presence of risedronate. During the incubation, testing and evaluation the standard protocols were used. MTT and TUNEL assays were employed to estimate changes in cell viability and percentage of apoptosis. We found that a combination of doxorubicin and cisplatin with risedronate sodium produced the strongest cytotoxic effects in both cell cultures. The cytotoxicity against both types of *osteosarcoma* was concentration-dependent and the cytostatic drugs and risedronate sodium were found to act synergistically.

**Abstract:**

The study discusses in vitro cytotoxicity of a combination of cytostatic drugs (doxorubicin, cisplatin, carboplatin, etoposide) and risedronate sodium against canine and human *osteosarcoma* (D-17 and U-2 OS). Standard protocols were used for the preparation of cell cultures and evaluation of their viability and apoptosis. MTT assay assessed the culture viability and EC_50_, while the apoptotic effect of the drugs was checked with a TUNEL assay. Doxorubicin alone showed the strongest cytotoxicity against D-17 (0.056 ± 0.019 µg/mL) and U-2 OS (0.051 ± 0.003 µg/mL), while the lowest cytotoxicity was observed for carboplatin (D-17, 6.45 ± 0.2 µg/mL and U2-OS, 27.5 ± 2.3 µg/mL). Risedronate sodium at 100, 10 and 1 µg/mL lowered viability in OS cell lines by 53.38 ± 1.46 and 49.56 ± 0.7%, 97.08 ± 3.32 and 74.92 ± 4.01%, and 102.67 ± 3.56 and 94.56 ± 3.52%, respectively. In all analyzed drug combinations, risedronate sodium significantly (* *p* < 0.05) increased the cytotoxicity against tested *osteosarcoma* cell lines. The decrease in cell viability caused by the studied compound combinations was weaker in canine than in human cell cultures. A combination of doxorubicin (all concentrations), cisplatin (1 µg/mL) and etoposide (1 µg/mL) with 100 µg/mL of risedronate sodium significantly improved the cytotoxicity of the drugs against canine and human *osteosarcoma*. Administration of carboplatin (1 µg/mL) and risedronate sodium (100 µg/mL), compared to carboplatin *per se*, produced no significant differences in cytotoxicity against the D-17 cell culture but significantly enhanced cytotoxicity in the U-2 OS line. The strongest apoptosis in both lines was detected for 0.01 µg/mL doxorubicin combined with 100 µg/mL risedronate sodium or 1 µg/mL cisplatin and 100 µg/mL risedronate sodium. In all combinations, the tested compounds revealed a synergistic mechanism of action.

## 1. Introduction

*Osteosarcoma* (OS) is the most common primary bone tumor, diagnosed in 80–85% of canine patients with bone neoplasms. The disease is an important problem of human and veterinary oncology. This neoplasm is a primary bone tissue malignancy of mesenchymal origin and highly diverse histopathological structure. Considering all diagnosed neoplasms in dogs, the prevalence of OS is moderate. It occurs usually in adult large and giant breed dogs (2–15 years old) [1,2,3,4,5,6]. From the biomechanical perspective, canine OS is the most often diagnosed in the long bones, i.e., the structures of the locomotor system that are the most exposed to intense mechanical stresses and associated micro-damage. The animal size can significantly affect the physical forces that act on the bone’s internal structure. They may affect both the muscle attachment points and the entire organ serving as a part of the skeletal system. Moreover, OS etiology has a genetic background [7].

The histopathology, location and predisposing factors of canine and human OS are highly similar [8]. Children are more predisposed, with a peak incidence at the age of 16, because of accelerated bone tissue turnover. The second OS peak incidence can be seen in the elderly, when Paget’s disease, radiation or bone infarction are the most common causes [9,10]. A routine canine and human OS therapy involves a surgical intervention including the tumor tissue resection or limb amputation. The postoperative adjuvant chemotherapy or typical course of chemotherapy is the most frequently used pharmacological treatment [11].

### 1.1. Bisphosphonates

The newest generation of bisphosphonates (risedronate sodium) have the strongest affinity for tissues of the skeletal system. Risedronate molecules accumulate in the bone matrix. They do not penetrate the cell cytoplasm but inhibit hydroxyapatite degradation and decrease osteoclast activity by inhibition of the mevalonate pathway leading to apoptosis [12]. The accessible literature lacks wider information on the in vitro cytotoxicity of risedronate sodium, even though it is commonly administrated in Paget’s disease and osteoporosis. So far, the clinical evaluation of its cytotoxicity was carried out during OS therapy [13]. The pharmacological effects of bisphosphonate administration include pain relief, as well as inhibition of osteolytic and proliferation activity of OS cells [14,15]. The combination of risedronate sodium with cytostatic drugs routinely used in OS therapy (cisplatin, carboplatin) and other cytotoxic compounds (doxorubicin, etoposide) can be a valuable contribution to studies on the response of both tested cell cultures.

### 1.2. Cytotoxic Drugs

Platinum derivatives chosen for this study (cisplatin and carboplatin) are inorganic, hydrophilic and DNA alkylating compounds. Cisplatin or carboplatin are cytostatics routinely used in humans and animals. The mechanism of their cytotoxic action consists of binding to numerous endogenous compounds with nucleophilic functional groups (e.g., glutathione, methionine, metallothionein) that are typical not only for DNA but also for RNA, enzymes or hormones [16]. Cisplatin bound to other substances is inactive. Its mechanism of action is based on transcription and translation inhibition. Then, p53 or other controlling proteins identify DNA damage and initialize the cell apoptosis. Cisplatin shows the highest activity in the S-phase and the lowest in the G0-phase of the cell cycle. Drug resistance may occur. Its mechanisms include reduced transmembrane transportation, lower intracellular accumulation, higher DNA repair ability, inactivation by binding with thiourea or glutathione and synthesis of apoptosis inhibitors (e.g., survivin) [17].

Carboplatin is a derivative of cisplatin with similar pharmacological characteristics and a similar mechanism of action.

### 1.3. Herbal Medicines

Etoposide is a plant based semi-synthetic derivative of podophyllotoxin extracted from *Podophyllum* sp. The drug is used in the medical treatment of humans and dogs. Its pharmacodynamics are not fully elucidated but etoposide affects the activity of topoisomerase II and subsequently causes DNA damage [18]. This may be augmented by free radicals, the production of which is induced by the drug. The final result of DNA damage is cell apoptosis. As the proliferation rate of neoplastic cells is higher than in normal tissues, the cytotoxic effect of etoposide is stronger in neoplasms. The highest activity of etoposide is noted in the S-phase and at the beginning of the G2-phase, which prevents cells from reaching the M-phase [19]. Resistance to etoposide may be due to the lowered expression of topoisomerase II and structural changes in the enzyme. Other mechanisms include the increased repair ability of DNA and greater etoposide evacuation from cells (higher concentration of proteins responsible for multi-drug resistance, e.g., glycoprotein P).

### 1.4. Anthracycline Antibiotics 

Doxorubicin is a compound of natural origin isolated from *Streptomyces peucetius v. cesius*. The drug is used in canine, feline, human and ferret anticancer therapy. Its mechanism of action is based on doxorubicin incorporation into the DNA helix and inhibition of topoisomerase II activity [20]. Additionally, the drug increases the level of free radicals resulting in cell membrane, DNA and protein disintegration. DNA damage activates control proteins, e.g., p53, and induces cell apoptosis. Doxorubicin affects the cell cycle but does not aim for a specific phase. The drug resistance is realized by its greater evacuation from the cell (higher concentration of proteins responsible for multi-drug resistance, e.g., glycoprotein P). Other responsible mechanisms are decreased activity or mutation of topoisomerase II, higher activity of glutathione peroxidase or increased DNA repair ability.

The objective of our study was to assess the pharmacological interaction between risedronate sodium and selected cytostatic drugs with cytotoxic effects against *osteosarcoma* (cisplatin, carboplatin) or other neoplasms (doxorubicin, etoposide). Risedronate sodium combined with those agents may change the cytotoxic effects observed in the investigated cell cultures, either by synergy or addition.

## 2. Materials and Methods

### 2.1. Cell Cultures

Culture flasks (25 cm^2^) (TPP, Trasadingen, Switzerland) with OS cell lines (D-17 and U-2 OS; ATCC, Manassas, VA, USA) were incubated in standard conditions (37 °C, 5% constant flow of CO_2_). Supplementation of an Eagle’s Minimum Essential Medium (EMEM) and a McCoy’s 5A culture medium (ATCC, Manassas, VA, USA) with 10% fetal bovine serum (FBS) (Sigma-Aldrich, Burlington, MA, USA), 4 nM L-glutamine (Sigma-Aldrich, Burlington, MA, USA), 100 U/mL penicillin, and 100 µg/mL streptomycin (Sigma-Aldrich, Taufkirchen, Germany) was used.

Trypsin solution (Sigma-Aldrich, Burlington, MA, USA) was added to OS cell cultures to peel them off the flask bottom. The cell suspension (10 µL) was mixed with 10 µL of Trypan blue (Sigma-Aldrich, Burlington, MA, USA) and 80 µL of PBS (Institute of Immunology and Experimental Therapy, Polish Academy of Sciences, Wrocław, Poland). The number of cells was calculated by a hemocytometer.

### 2.2. Tested Compounds

The drugs (Sigma-Aldrich, Taufkirchen, Germany) were tested at the following concentrations: doxorubicin 1, 0.5, 0.1, 0.05, 0.01, 0.005 and 0.001 µg/mL; cisplatin and carboplatin 50, 20, 10, 5, 1, 0.5, 0.1 and 0.05 µg/mL; etoposide 10, 5, 2.5, 1, 0.5, 0.1, 0.05, 0.025, 0.01, 0.005 and 0.0025 µg/mL; and risedronate sodium 300, 150, 100, 30, 15, 10, 3, 1.5, 1, 0.3 and 0.15 µg/mL.

### 2.3. Cell Viability

The cell cultures were incubated for 24 h in 96 well culture plates (TPP, Trasadingen, Switzerland) at a density of 3 × 10^3^/100 µL per well, under the same conditions as in the flasks. Then the medium (EMEM and McCoy’s 5A) was changed and the cultures were exposed to the studied compounds alone (as mentioned above) or in combinations (Table 1) for 72 h. The drug concentrations were chosen according to the literature, maximum concentration of the drug in serum and maximum non-cytotoxic concentration of diluents (ethanol and DMSO) [21,22,23,24,25,26,27,28]. Negative control included untreated cells, and mitomycin C (Sigma-Aldrich, Burlington, MA, USA) at 0.1 µg/mL was used as a positive control. Following the incubation with the tested compounds, 20 µL of 5 mg/mL MTT solution (Sigma-Aldrich, Burlington, MA, USA) were added into each well and further incubated for 4 h. Subsequently, 80 µL of a lysis buffer, 225 mL of dimethylformamide (Sigma-Aldrich, Taufkirchen, Germany), 6.5 g of sodium dodecyl sulfate (Sigma-Aldrich, Burlington, MA, USA) and 275 mL of distilled water were applied to destroy the cell membranes.

After 24 h incubation the absorbance was measured with a spectrophotometer (Multiscan GO, Thermo Scientific, Waltham, MA, USA). The viability of cells was estimated according to the following formula [29]:

$$\mathrm{Viability}\;[\%]=100\%\;\times \frac{meanopticaldensityoftreatedcells}{meanopticaldensityofuntreatedcells}$$

Four independent repetitions were carried out for the individual substances and the mean value and EC_50_ were computed. For drug combinations only the mean value was calculated.

The concentrations that decreased the cell viability by more than 50% (values lower than EC_50_), were chosen for further tests. This procedure allowed for confirmation of pharmacological interactions between the tested substances. The results underwent a statistical analysis. The combinations of drug concentrations selected for further analysis are shown in Table 1.

### 2.4. Estimation of Cell Apoptosis

The share of apoptotic cells was estimated using the ApopTag^®^ Peroxidase In Situ Apoptosis Detection Kit. The kit consisted of an Equilibration Buffer, Working Strength TdT Enzyme, Stop/Wash Buffer, Anti-digoxigenin Peroxidase Conjugate and DAB Peroxidase Substrate (Merck Millipore, Darmstadt, Germany).

The tested cultures of OS cell lines at a concentration of 2 × 10^4^ cells/40 µL of the medium were placed in 10-well polytetrafluoroethylene coated slides (Thermo Scientific, USA) and incubated for 24 h. Then, the tested compounds were added after the medium removal (Table 2). After that, the cells were fixed in 1% formaldehyde (POCH, Gliwice, Poland), pH 7.4, for 10 min at room temperature (RT). The slides were washed two times (5 min each) in PBS and incubated in cold ethanol and acetic acid (1:1) for 5 min at −20°C. The procedure not only fixed the cells but also allowed for cell membrane permeability. The slides were then rinsed in PBS (Institute of Immunology and Experimental Therapy, Polish Academy of Sciences, Wrocław, Poland) twice for 5 min and inserted into a 3% hydrogen dioxide solution (POCH, Gliwice, Poland) to block endogenous peroxidase. After another washing with PBS (2 × 5 min), Equilibration Buffer was added (75 µL/5 cm^2^) and the cells were incubated for about one minute. Directly after the buffer removal, the cell cultures with 55 µL/5 cm^2^ of Working Strength TdT Enzyme were incubated at 37°C, in a moist chamber, for 1 h. Next, the slides were put into Stop/Wash Buffer for 10 min and subsequently rinsed in PBS (3 × 1 min). At the next step, 65 μL/5 cm^2^ of Anti-digoxigenin Peroxidase Conjugate was added to each well and the slides were incubated in the moist chamber for 30 min at RT. After another cycle of rinsing in PBS (4 × 2 min), 75 μL/5 cm^2^ of DAB Peroxidase Substrate was added and the hydrophobic slides were incubated at RT for 10 min. Finally, the slides were washed in distilled water (3 × 1 min) and incubated for 5 min in bi-distilled water. The cell nuclei were stained with 1% haematoxylin (Sigma-Aldrich, Taufkirchen, Germany) and dehydrated in 70% ethanol for 30 s and in xylene for 30 s (Stanlab, Lublin, Poland). The final stage of the procedure involved mounting a cover glass on each slide using DPX (Thermo Scientific, Waltham, MA, USA).

**Figure 1:**
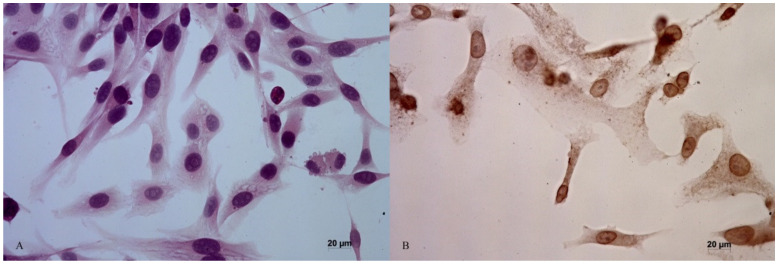
Non-apoptotic (**A**) and apoptotic (**B**) D-17 cell cultures observed in optical microscopy, 40×. B-brown pigment marks apoptotic cells.

The light microscope Olympus BX53 (Olympus, Tokyo, Japan) was used to estimate the percentage of apoptotic cells and to count them (5 fields of vision, magnification 40×). The results are presented as the mean for each field of vision (Figure 1). The procedure was carried out independently by two experienced researchers.

### 2.5. Statistical Analysis

The results were analyzed using the StatisticaPL 10.0 package (StatSoft, Kraków, Poland). The normal distribution of values was tested using the Shapiro-Wilk test, and comparison of drug combinations was carried out using the Kruskal-Wallis analysis. The results on canine and human OS were compared with the Mann-Whitney U test. The Spearman’s correlation coefficients were employed. Statistical significance was assumed at *p* = 0.05.

## 3. Results

### 3.1. Cytostatic Drugs Alone

The cytostatic drugs chosen for this study are routinely used in canine and human oncology. Doxorubicin showed the strongest cytotoxicity against OS cell cultures (D-17 and U-2 OS), as its EC_50_ reached 0.056 ± 0.019 µg/mL and 0.051 ± 0.003 µg/mL, respectively. Of the tested drugs carboplatin showed the weakest cytotoxicity, with EC_50_ 6.45 ± 0.2 µg/mL and 27.5 ± 2.3 µg/mL for the canine and human OS cell line, respectively. Only one form of the third generation of bisphosphonates was chosen for this study. Risedronate sodium exhibited the weakest cytotoxic activity among all tested compounds (Table 3).

### 3.2. Cytostatic Drugs Combined with Risedronate Sodium

**Figure 2:**
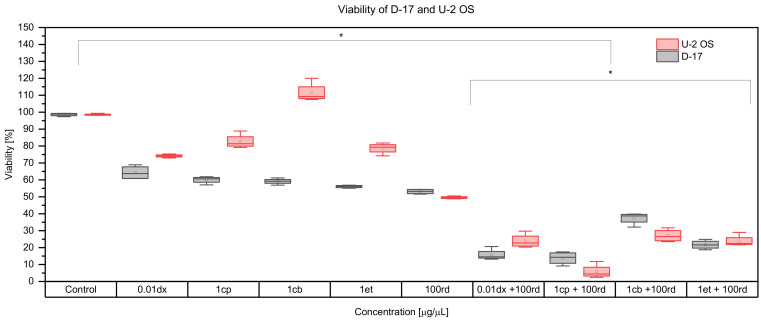
The effect of risedronate sodium on cytostatic activity of doxorubicin, cisplatin, carboplatin, etoposide *per se* and in combined administration. dx—doxorubicin, cp—cisplatin, cb—carboplatin, et—etoposide, rd—risedronate sodium, * (*p* < 0.05).

The combined administration of selected doses of doxorubicin, cisplatin, carboplatin, etoposide and risedronate sodium in both studied OS cell lines is presented in Figure 2. Risedronate sodium enhanced the cytotoxicity of doxorubicin (*p* < 0.05) and at its dose of 100 µg/mL the cytotoxic effect of doxorubicin was the strongest (*p* < 0.05) in both lines. The canine cell line turned out to be more susceptible to the combination of risedronate sodium and doxorubicin than the human one. Cisplatin together with risedronate sodium exerted a significant (*p* < 0.05) cytotoxicity against the tested cell lines, which was not the case when the substances were used at the same doses but separately. Combined use of 1 µg/mL cisplatin and 100 µg/mL risedronate sodium resulted in the strongest cytotoxic effect and the viability of canine and human cells was 13.44 ± 3.58 and 5.65 ± 4.28%, respectively. We found significant differences in the cytotoxicity between the canine and human OS cell lines after joined use of carboplatin and risedronate sodium. The viability of canine OS cells treated with 1 µg/mL carboplatin and 100 µg/mL risedronate sodium reached 37.2 ± 3.75%. Even though it was lower than after separate administration of the drugs, the difference was not of statistical significance (*p* > 0.05). In the case of the U-2 OS cell line, the combination of 1 µg/mL cisplatin and 100 µg/mL risedronate sodium in comparison with separate administration of the drugs showed a significant difference and cell viability was 27.75 ± 3.84% (*p* < 0.05). The tested cell lines were more susceptible to the combination etoposide and risedronate sodium than to their separate administration. The cytotoxic effect depended rather on the concentration of risedronate sodium than that of etoposide. The strongest cytotoxicity was observed for 100 µg/mL of risedronate sodium and 1 µg/mL of etoposide. The viability of the canine OS cell line was 21.84 ± 2.99%, and highly similar to that of the human OS cell line that reached 23.91 ± 4.14%. These values were 50% lower than for the drugs used alone at the same concentrations.

### 3.3. The Apoptotic Effect of Combined Use of Risedronate Sodium and Cytostatic Drugs 

**Figure 3:**
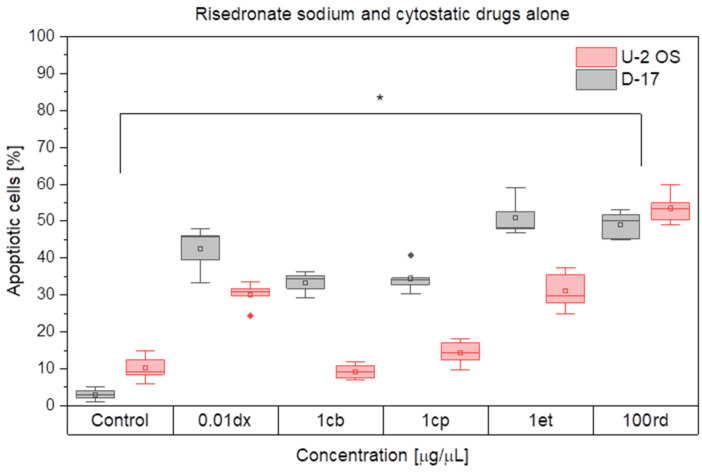
The effect of cytostatic drugs and risedronate sodium *per se* on the cell apoptosis in the canine and human *osteosarcoma* cell lines. dx—doxorubicin, cp—cisplatin, cb—carboplatin, et—etoposide, rd—risedronate sodium, rhombus—outliers, * (*p* < 0.05).

**Figure 4:**
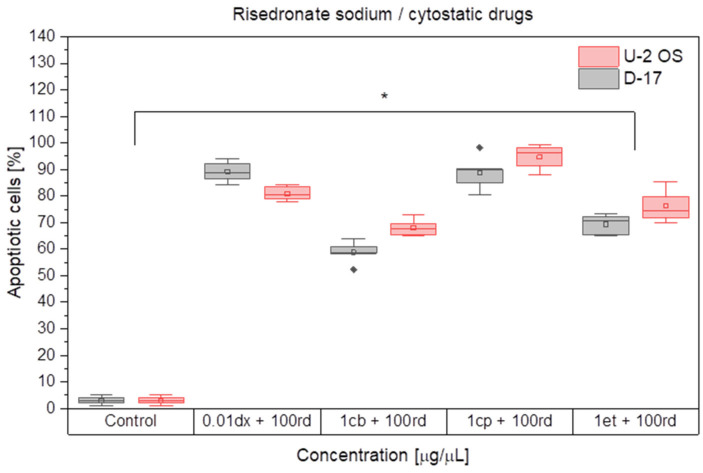
The effect of combinations of cytostatic drugs and risedronate sodium on the apoptosis in canine and human *osteosarcoma* cell lines. dx—doxorubicin, cp—cisplatin, cb—carboplatin, et—etoposide, rd—risedronate sodium, rhombus—outliers, * (*p* < 0.05).

The study demonstrated that the pro-apoptotic effect in D-17 and U-2 OS cell cultures after combined administration of the cytotoxic drugs (doxorubicin, cisplatin, carboplatin and etoposide) and risedronate sodium may be described as synergy. The strongest apoptosis in both cell cultures was caused by 0.01 µg/mL doxorubicin combined with 100 µg/mL risedronate sodium or 1 µg/mL cisplatin and the same dose of risedronate sodium. The apoptosis rate in the cultures treated with these combinations was 89.21 ± 4.07% and 88.70 ± 6.67% for the canine OS cell line, and 80.91 ± 2.83% and 94.66 ± 4.86% for the human OS cell line, respectively (*p* < 0.05) (Figure 3 and Figure 4).

## 4. Discussion

The study investigated the cytotoxicity of cisplatin, carboplatin, doxorubicin and etoposide against D-17 and U-2 OS cell lines. All the investigated compounds are routinely used in oncological therapy of neoplasms in humans and animals [30,31]. The comparison of tested compounds and their combinations is needed for statistical analysis of the achieved results to define the potential synergistic or antagonistic pharmacological effect. Of the tested compounds, the strongest cytotoxicity was noted for cisplatin in both OS cell lines (*p* < 0.05). This was also particularly visible for viability that dropped to 0.00% for canine and 0.01% and 1.03% for human OS cells, following administration of cisplatin at 50 and 20 µg/mL [32].

Moreover, Poradowski [32] showed considerably strong cytotoxicity and decreased viability of both cell cultures after doxorubicin administration. Mentioned studies proved that doxorubicin at 1µg/mL caused the viability to decrease to 17.81 ± 6.55% and 9.23 ± 0.61% in canine and human lines, respectively [32]. In other words, low viability of the tested OS cell lines exposed to cisplatin and doxorubicin, together with low value of EC_50_ in both cases, proved similar susceptibility of the evaluated cell cultures to these cytostatic drugs. The two remaining compounds, carboplatin and etoposide, exhibited lower cytotoxicity, as the viability of the tested cell lines reached 34.25 ± 3.92 and 47.04 ± 1.43% and 10.08 ± 5.26 and 24.44 ± 3.83% [32], respectively. Similar observations were carried out during statistical analysis of carboplatin and etoposide combined use.

Carboplatin showed higher EC_50_, and the cells of canine OS seemed more susceptible to this drug (EC_50_ = 6.45 ± 0.2 µg/mL) than human OS cells (EC_50_ = 27.5 ± 2.3 µg/mL) (*p* < 0.05). A reverse pattern was observed for the canine and human OS after treatment with etoposide, where human cells showed greater susceptibility (EC_50_= 2.72 ± 0.51 µg/mL) than canine ones (EC_50_= 6.27 ± 0.31 µg/mL) (*p* < 0.05). Following treatment with carboplatin and etoposide, cell viability and EC_50_ were lower than after the administration of cisplatin and doxorubicin, which proved stronger activity of the latter two drugs against the evaluated cell lines. Several protocols of OS treatment in humans and animals are based on the combined administration of cisplatin and doxorubicin or on a synergy between these and other cytotoxic drugs (i.e., methotrexate or ifosfamide) [33,34].

Summarizing, cisplatin alone showed the strongest cytotoxicity against both human and canine OS cell lines, but at higher doses than doxorubicin. The latter was also twice as effective against human than canine OS cell lines. Finally, carboplatin and etoposide brought about the weakest cytotoxic effect, with carboplatin being more effective toward canine and etoposide toward human OS cell lines. The last evaluated compound was risedronate sodium (a bisphosphonate of the third generation), used for treating bone tissue metabolic disorders in humans. The potential cytotoxicity of bisphosphonates suggests their possible use in supporting the activity of cytotoxic drugs in the therapy of neoplasms, including myeloma multiplex or OS [29,35,36,37]. Viability analysis of D-17 and U-2 OS cell lines exposed to a wide range of risedronate sodium concentrations proved its concentration-dependent cytotoxicity (*p* < 0.05). Risedronate sodium at 100, 10 and 1 µg/mL decreased the viability of canine and human *osteosarcoma* cell lines to 53.38 ± 1.46 and 49.56 ± 0.7%, 97.08 ± 3.32 and 74.92 ± 4.01%, 100 ± 3.56 and 94.56 ± 3.52%, respectively. The viability of the investigated cell lines and the concentration of risedronate sodium showed a negative correlation that reached *p* < 0.05, r = 0.42 and *p* < 0.05, r = 48 for D-17 and U-2 OS, respectively. A comparison of EC_50_ for canine (D-17; EC_50_= 144.83 ± 6.22 µg/mL) and human (U-2 OS; EC_50_= 98.1 ± 5.4 µg/mL) OS cell line demonstrated that human OS cells were more susceptible to risedronate sodium than canine OS cell cultures (*p* < 0.05) [13]. Similar studies on the viability of U-2 OS and SaOS-2 after risedronate sodium administration at 0, 0.1, 1, and 10 µM/mL (≈ 0, 0.03, 0.3, 3 µg/L revealed no statistical significance [37]. Earlier work by Poradowski et al. [13] reported on the strong cytotoxicity of risedronate sodium but only at concentrations close to 100 µg/mL. Taken together with Poirier’s et al. [29] studies on zoledronate, it seems justified to claim that bisphosphonates show strong cytotoxicity against D-17, Abrams, MG-63 and SaOS-2 cell lines. Their cytotoxicity depends on the drug concentration and is more evident at higher doses.

A study by Farese at al. [35] described the anticancer effect of the second and third generation bisphosphonates. Similar results for alendronate sodium were reported by Poirier et al. [29]. Our statistical analysis of viability and EC_50_ in the cell cultures exposed to all tested compounds indicated the strongest cytotoxicity of doxorubicin and cisplatin against canine and human OS (0.056 ± 0.019 and 0.051 ± 0.003 µg/mL and 2.35 ± 0.43 and 2.38 ± 0.43 µg/mL for *p* < 0.05). Carboplatin and etoposide exhibited weaker cytotoxic effect in canine OS cell cultures. Human OS was the most resistant to carboplatin (EC_50_= 27.5 ± 2.3 µg/mL) and etoposide and cisplatin cytotoxicity in this line was comparable with EC_50_= 2.72 ± 0.51 and 2.38 ± 0.43 µg/mL, respectively. Risedronate sodium alone exerted a significant cytotoxic effect only at higher doses and the cell susceptibility was higher in the human OS line.

The study aimed at investigating a combined activity of risedronate sodium and other cytotoxic drugs in *osteosarcoma* cell lines (U-2 OS and D-17). In our earlier work [13], we demonstrated the cytotoxic effects of risedronate sodium combined with meloxicam, and concluded that the effects were due to a synergistic activity of both compounds. Moreover, the combination of risedronate sodium and meloxicam was found more detrimental to the U-2 OS than the D-17 line (*p* < 0.05) [13]. Therefore, more detailed studies on the interactions between risedronate sodium and chosen cytostatic drugs seems a promising research direction.

The combinations of the cytostatic drugs and risedronate sodium exhibited strong anticancer activity in the tested *osteosarcoma* cell lines. Similarly, as in an earlier study by Poradowski et al. [13], potentialization of the cytostatic drugs by combining them with risedronate sodium was statistically significant and may be interpreted as synergy. A study by Murayama et al. [38] in U-2 OS, SaOS-2 and MG63 cell lines reported similar observations for risedronate sodium, doxorubicin, carboplatin, vincristine and etoposide. Our statistical analysis confirmed the strongest synergy for cisplatin combined with risedronate sodium against the D-17 cell line. The synergy between other bisphosphonates (zoledronate sodium, clodronate disodium, alendronate sodium, pamidronate sodium) and doxorubicin or vincristine was proved by Hafeman et al. [39] in an established cell line (DH82) and two primary cell cultures (canine malignant histiocytosis). It seems justified to hypothesize that, similar to the combination of risedronate sodium and meloxicam, the mentioned bisphosphonate also acted synergistically, especially with cisplatin. Moreover, the effects of this synergy were stronger in the canine OS cell line.

Our study revealed a very strong apoptotic effect of risedronate sodium in D-17 and U-2 OS cell lines. Murayama et al. [38] also observed apoptotic activity of risedronate sodium in a murine *osteosarcoma* (LM-8) cell culture and proved its concentration dependence. Apart from risedronate sodium, other bisphosphonates, such as zoledronate sodium and alendronate sodium, strongly promote apoptosis in both tested cell lines [29]. A considerable apoptotic rate of over 80% was observed in canine and human OS cell cultures treated with risedronate sodium and doxorubicin or cisplatin combinations. The enhanced apoptotic rate was brought about by a combination of risedronate sodium with carboplatin or etoposide with a significance of *p* < 0.05. Still, the activity of these combinations was lower than that presented by doxorubicin and cisplatin combined with risedronate sodium. The pro-apoptotic activity of all compound combinations confirmed the results achieved in the MTT assay.

The study results seem to support the hypothesis that both pro-apoptotic and cytotoxic activity of the investigated cytostatic drugs can be increased by risedronate sodium. A similar synergy between zoledronate sodium and doxorubicin was reported by Neville-Webbe et al. [40] in the established human cell lines of breast cancer (MCF-7 and MDA-MB-436) and prostate cancer (PC3). The impact of the investigated combinations on normal human or canine cell lines was not evaluated in this study. The promising synergy, observed in cytotoxicity and the pro-apoptotic activity of cytostatic drugs promoted in vitro by risedronate sodium needs further studies to exclude a possible increase in these properties in cell lines used as normal tissue models (e.g., NHDF, MDCK, HEK-293).

Finally, all results achieved in vitro must be verified during in vivo experiments. The concentrations of the investigated compound were chosen on the basis of accessible literature. Even though they may be achieved in the blood plasma of living animals or humans, only the studies in experimental animals can confirm or disprove the synergistic effects of risedronate sodium combined with cytostatic drugs.

## 5. Conclusions

The supporting role of risedronate sodium in the cytotoxic effects provided by drugs used in the treatment of OS and other neoplasms was demonstrated in vitro in D-17 and U-2 OS cell lines. Our analysis can confirmed the hypothesis of a synergistic character of these combinations of cytostatic compounds. Further studies on normal human and canine cell lines and subsequent in vivo experiments are needed to verify the presented in vitro outcomes.

## Figures and Tables

**Table 1:** Tested drug combinations: dx—doxorubicin, cp—cisplatin, cb—carboplatin, et—etoposide, rd—risedronate sodium.

Combinations [µg/mL]			
1dx + 100rd	1cp + 100rd	1cb + 100rd	1et + 100rd
1dx + 10rd	1cp + 10rd	1cb + 10rd	1et + 10rd
1dx + 1rd	1cp + 1rd	1cb + 1rd	1et + 1rd
0.1dx + 100rd	0.1cp + 100rda	0.1cb + 100rd	0.1et + 100rd
0.1dx + 10rd	0.1cp + 10rd	0.1cb + 10rd	0.1et + 10rd
0.1dx + 1rd	0.1cp + 1rd	0.1cb + 1rd	0.1et + 1rd
0.01dx + 100rd	0.01cp + 100rd	0.01cb + 100rd	0.01et + 100rd
0.01dx + 10rd	0.01cp + 10rd	0.01cb + 10rd	0.01et + 10rd
0.01dx + 1rd	0.01cp + 1rd	0.01cb + 1rd	0.01et + 1rd
0.005dx + 100rd			
0.005dx + 10rd			
0.005dx + 1rd			
0.001dx + 100rd			
0.001dx + 10rd			
0.001dx + 1rd			

**Table 2:** Concentrations selected for the assessment of apoptosis. dx-doxorubicin, cp-cisplatin, cb-carboplatin, et-etoposide, rd-risedronate sodium.

Combinations [µg/mL]			
0.01dx + 100rd	1cp + 100rd	1cb +100rd	1et +100rd

**Table 3:** EC_50_ values for selected cytostatic drugs and risedronate sodium.

EC_50_ [µg/mL]		
	D-17 cell line	U-2 OS cell line
doxorubicin	0.056 ± 0.019 µg/mL	0.051 ± 0.003 µg/mL
cisplatin	2.35 ± 0.43 µg/mL	2.38 ± 0.43 µg/mL
carboplatin	6.45 ± 0.2 µg/mL	27.5 ± 2.3 µg/mL
etoposide	6.27 ± 0.31 µg/mL	2.72 ± 0.51 µg/mL
risedronate sodium	144.83 ± 6.22 µg/mL	98.1 ± 5.4 µg/mL

## Data Availability

Not applicable.
